# Real-time predictors of food parenting practices and child eating behaviors in racially/ethnically diverse families

**DOI:** 10.1186/s12966-023-01476-4

**Published:** 2023-07-11

**Authors:** Jerica M. Berge, Angela R. Fertig, Amanda Trofholz, Junia N. de Brito

**Affiliations:** 1 Department of Family Medicine and Community Health, University of Minnesota Medical School, 717 Delaware Street SE, Room 425, Minneapolis, MN 55414 USA; 2 Humphrey School of Public Affairs, University of Minnesota Twin Cities, Minneapolis, MN USA

**Keywords:** Stress, Depressive symptoms, Ecological momentary assessment, Food parenting practices, Child eating behaviors, Food insecurity

## Abstract

**Background:**

Prior research has shown associations between controlling food parenting practices (e.g., pressure-to-eat, restriction) and factors that increase risk for cardiovascular disease in children (e.g., low diet quality, obesity). This study aimed to examine associations between real-time parental stress and depressed mood, food parenting practices, and child eating behaviors in a longitudinal cohort study.

**Methods:**

Children ages 5–9 years and their families (*n* = 631) from six racial/ethnic groups (African American, Hispanic, Hmong, Native American, Somali/Ethiopian, White) were recruited for this study through primary care clinics in a large metromolitan area in the US (Minneapolis/St. Paul, MN) in 2016–2019. Ecological momentary assessment was carried out over seven days with parents at two time points, 18 months apart. Adjusted associations between morning stress and depressed mood of parents on food parenting practices and child eating behaviors at the evening meal were examined. Interactions tested whether food security, race/ethnicity and child sex moderated associations.

**Results:**

High levels of parental stress and depressed mood experienced earlier in the day were associated with controlling food parenting practices and child food fussiness at dinner the same night. Results were dependent on food security status, race/ethnicity, and child sex.

**Conclusions:**

Health care professionals may want to consider, or continue, screening parents for stress, depression, and food insecurity during well-child visits and discuss the influence these factors may have on food parenting practices and child eating behaviors. Future research should use real-time interventions such as ecological momentary intervention to reduce parental stress and depressed mood to promote healthy food parenting practices and child eating behaviors.

**Supplementary Information:**

The online version contains supplementary material available at 10.1186/s12966-023-01476-4.

## Background

Parents influence child eating behaviors both through “how” (i.e., food parenting) and “what” (i.e., food served at meals) they feed their children. Controlling food parenting practices (“how” parents feed their children), such as restriction and pressure-to-eat are associated with overweight [1–4], low diet quality [1–3, 5], lower satiety responsiveness [2, 3, 6, 7] and unhealthly weight control behaviors [8] in children. Serving less healthful foods (“what” parents feed their children) at family meals (e.g., energy-dense foods, high-fat foods, sugar-sweetened beverages) is associated with low diet quality and overweight status in children [9–12] In addition, child eating behaviors such as food fussiness (i.e., picky eating) are associated with consuming fewer fruits and vegetables [13]. Understanding real-time predictors (e.g., stress, depressed mood) of food parenting practices and child eating behaviors is important for developing interventions that can be delivered in the moment when these behaviors are occurring to reduce future cardiovascular disease (CVD) risk in children [14, 15].

Two potential modifiable predictors of food parenting and child eating behaviors include parental stress and depressed mood [16]. Prior cross-sectional studies have shown that maternal depressed mood was associated with increased risk of engaging in pressure-to-eat [17] food parenting practices and serving less healthy foods at family meals [18]. One prior study using ecological momentary assessment showed that the combination of high parental stress and depressed mood earlier in the day was associated with increased risk of parents engaging in pressure-to-eat feeding practices at the meal the same evening [19]. With regard to child eating behaviors, previous research has shown that food parenting practices influence child eating behaviors [1, 7, 20, 21] however, we are unaware of any prior research examining associations between parental stress and child eating behaviors.

While these prior studies suggest associations between parental stress, depressed mood, food parenting practices and child eating behaviors, there are limitations with these previous studies. One limitation is that the majority of prior studies use static, self-reported measures, which are prone to social desirability bias and assume that food parenting practices and child eating behaviors do not fluctuate over time and context. Another limitation is the lack of large samples from racially/ethnically diverse backgrounds, which is important, given the high prevalence of obesity and CVD in families from diverse backgrounds [14, 22–24], due to social marginalization of racialized families [25].

Contextual and social constructs such as food insecurity are important to consider when examining real-time predictors of food parenting practices and child eating behaviors. Food insecurity creates an environment in which parents engage in food parenting practices when food is scarce [26, 27] which may alter their typical food parenting practices [28] Our own pilot study showed that high levels of parental stress and depressed mood experienced earlier in the day within food insecure households were associated with using restrictive feeding practices and serving more store bought foods at the evening meal the same night [29] Furthermore, child eating behaviors may be altered in a food insecure household [30, 31]. For example, a child may become more or less food responsive because of the lack of food due to fewer food choices available, or parents may become less responsive to their child’s food requests.

The current study will build on and extend prior research by examining longitudinal real-time associations between parent stress, depressed mood, food parenting practices, and child eating behaviors by using ecological momentary assessment (EMA) of both exposure variables (i.e., parent stress, depressed mood) and outcome variables (i.e., food parenting practices, healthfulness of foods served at dinner, child eating behaviors). The main hypotheses is that high parental stress and depressed mood earlier in the day will be associated with controlling food parenting practices, serving less healthful foods, and child food fussiness at dinner the same day and that there will be differences in these associations by race/ethnicity, child sex, and food security status. Identifying real-time predictors of food parenting and child eating behaviors will inform development of interventions that can intervene on these momentary behaviors and ultimately reduce risk for CVD over time.

## Methods

Data for the current study are from the *Family Matters* study [32], a longitudinal cohort study of children ages 5–9 (*n* = 1307) and their families from low-income and racially/ethnically diverse households. Data were collected via an online survey for the full sample and EMA data collection for a subsample (*n* = 631), both collected at two-time points 18-months apart (2016–2019 and 2018–2021). Between baseline and follow-up timepoints the sample retention rate was 96%.

### Recruitment

Parent/child dyads were recruited from primary care clinics in a large midwestern US metropolitan area (Minneapolis/St. Paul, Minnesota). Parents and children were eligible to participate in the study if: (1) the child was 5–9 years old; (2) the person completing the survey was the primary guardian of the 5–9 year old child; (3) the child lived with the parent/guardian more than 50% of the time; (4) the child was from one of the following racial/ethnic backgrounds: African American, Hispanic/Latinx, Hmong, Native American, Somali, Ethiopian, or White; and (5) the child had a body mass index greater than the 5^th^ percentile. The University of Minnesota’s Institutional Review Board Human Subjects Committee approved all protocols used in the study. All study materials (e.g., consent forms, survey questions) were translated into Hmong, Somali and Spanish. In-depth details about the study design, recruitment and eligibility criteria, sample, and measures are published elsewhere [32].

### EMA procedures

At the end of the online survey, parents who reported having three or more family meals per week were invited to participate in an optional EMA sub-study using their smartphone. Standardized EMA protocols based on best practice[33, 34] were used in developing EMA for the *Family Matters* study, including three signal-contingent surveys per day and an end-of-day survey. More details about EMA data collection are described in Table 1.

**Table 1:** Exposure, outcome, and moderation variables used in analyses

Construct	When Assessed	Question	Response Option	How Responses were Operationalized	Response Mean or Percent
**Collection of Ecological Momentary Assessment (EMA) Measures**					
**Signal Contingent EMA **[32, 34]	An automated EMA survey schedule was created so that signal-contingent surveys were sent to parents’ phones randomly throughout the day using the following criteria: (a) the first signal-contingent survey was sent at least an hour after the parent’s reported wake time; (b) the third signal-contingent survey was sent at least an hour after the parent’s reported dinner time; (c) the three signal-contingent surveys were spaced so that a minimum of one hour occurred between surveys. Parents were sent a text when signal-contingent surveys were available and had an hour to complete them, with reminders every 15 min.				
**End-of-day EMA** [32, 34]	End-of-day EMA surveys were sent to parents’ phones 90 min after their reported dinner time. Parents were sent a text when the end-of-day survey was available and had four hours to complete the survey. Parents completed seven days of EMA at each wave of data collection. A “complete” day for this study was defined as finishing at least 2 out of 3 signal-contingent surveys and the end-of-day survey. If parents did not finish a complete day, an extra day was added to their observation period. Once parents completed 7 days of EMA, they were automatically deactivated from the EMA system and were sent a $75 gift card by study staff. As a result of this protocol, all participants in the *Family Matters* EMA sample have 7 complete days of EMA surveys each wave of data collection.				
**Exposure Variables**					
**Parent stress** [35]	Signal contingent EMA surveys time-stamped before 12 p.m	On a scale from 0–10, with 0 being not stressed at all and 10 being very stressed, how would you rate your level of stress right now?	0, 1, 2, 3, 4, 5, 6, 7, 8, 9, 10	Parent stress, sources of stress, depressed mood, and depressive symptoms were summed. Multiple morning observations on the same day were averaged. To ease comparability across measures, all four measures were rescaled to range between 0 and 5 and were analyzed as continuous random variables	Mean = 1.20; SD = 1.32, *n* = 5,642 days 40.5% days had a morning stress rating of 0 25.1% days: 1–2 17.8% days: 3–4 9.4% days: 5–6 4.6% days: 7- 8 2.6% days: 8 +
**Sources of stress (asked if parent stress was > 0)** [36]		Since you woke up/since the last survey, have you experienced any of the following (select all that apply)?	Conflicts or arguments with family members or others | Demands from my family | Traffic/transportation problems | Concerns about news events | Problems with one's children| Concerns about medical treatment or health conditions (your own or other's) | Feeling conflicted over what to do | Worries about having enough money for the things I need or want | Too many things to do | Job dissatisfaction | Worries about upcoming family events (e.g., holidays, family vacation) | Unexpected change in plans | Childcare issues | Body image/weight concerns | Fatigue from lack of sleep | Some other problem or worry		Mean = 1.07; SD = 1.21, *n* = 5,642 days **Frequency of Sources of** **Stress Endorsed:** 41.5% of days no sources of stress were endorsed 30.2% days: 1–2 15.5% days: 3–4 7.5% days: 5–6 3.4% days: 7–8 1.8% days 8 + **Type of Stress Endorsed:** 9.7% of days “Conflicts or arguments with family members or others” was endorsed” 19.7% of days: “Demands from my family” 7.4% of days: “Traffic/transportation problems” 8.4% of days: “Concerns about news events” 8.0% of days: “Problems with one's children” 12.8% of days: “Concerns about medical treatment or health conditions (your own or other's)” 13.0% of days: “Feeling conflicted over what to do” 17.0% of days: “Worries about having enough money for the things I need or want” 24.5% of days: “Too many things to do” 6.7% of days: “Job dissatisfaction” 9.8% of days: “Worries about upcoming family events (e.g., holidays, family vacation)” 6.8% of days: “Unexpected change in plans” 4.1% of days: “Childcare issues” 10.8% of days: “Body image/weight concerns” 15.8% of days: “Fatigue from lack of sleep” 3.4% of days: “Some other problem or worry”
**Parent depressed mood** [32]		On a scale from 0–10, with 0 being not sad or depressed at all and 10 being very sad or depressed, how would you rate your level of sadness or depression RIGHT NOW?	0, 1, 2, 3, 4, 5, 6, 7, 8, 9, 10		Mean = 0.74; SD = 1.14, *n* = 5,642 days 59.6% days had a morning depressed mood rating of 0 21.4% days: 1–2 10.0% days: 3–4 5.4% days: 5–6 1.9% days: 7–8 1.8% days: 8 +
**Parent depressive symptoms** [37]		Since you woke up/since the last survey, have you felt: *Nervous? *Hopeless? *Restless or fidgety? *So depressed that nothing could cheer you up? *That everything was an effort? *Worthless?	Yes/No to each option		Mean = 0.35; SD = 0.94; *n* = 5,642 days **Frequency of Depressive** **Symptom Endorsed:** 82.6% of days no symptoms were endorsed 9.0% days: 1 4.0% days: 2 1.7% days: 3 0.8% days: 4 2.0% days: 5–6 **Type of Depressive** **Symptom Endorsed:** 9.4% of days: nervous was endorsed 4.5% of days: hopeless 7.7% of days: restless or fidgety 2.7% of days: depressed 7.5% of days: everything was an effort 3.7% of days: worthless
**Outcome Variables**					
**Pressure-to-eat** [38]	End-of-day EMA surveys time-stamped after 4 p.m	Did you have to encourage [child] to eat more food at this meal? Did you encourage [child] to eat more after s/he told you s/he was done? Did you make sure [child] ate all of the food on his/her plate before you let him/her stop?	Yes/No to each option	A positive answer to any of the three questions was coded as having engaged in pressuring practices that evening	24.9% of days; *n* = 5,642 days 14.8% encourage food 8.0% encourage after done 12.2% make sure clean plate
**Food restriction** [38]		Did you have to make sure [child] didn’t eat too much food at this meal? Did you have to make sure [child] didn’t eat too much of a certain food?	Yes/No to each option	If the respondent answered yes to either question, they were coded as having engaged in restrictive practices at that evening meal	6.2% of days; *n* = 5,642 days 3.3% restrict food 4.8% restrict specific food
**Instrumental feeding** [38]		Did you require [child] to finish certain foods before s/he could have other foods (e.g., dessert)?	Yes/No	A positive response was coded as having engaged in instrumental practices at that evening meal	12.5% of days; *n* = 5,642 days
**Foods served at dinner** [39]		Which best describes the type of food served? (Select all that apply)	Fast food / take-out (eaten at home or at a restaurant) | Pre-prepared foods (e.g. macaroni and cheese, frozen meals, cereal, chips) | Homemade / freshly prepared (include fresh fruits or vegetables here)	Three binary variables were created to capture foods served: fast food only, homemade only, or pre-prepared foods only	*n* = 5,642 days 14.9% of days served fast food/take out only 13.8% of days served pre-prepared meals only 63.1% of days served homemade meals only
**Child food fussiness** [40]		Did the child refuse to eat any of the food you offered him/her?	Yes/No	If the responded answered yes, the child was coded as having exhibited food fussiness that evening	9.0% of days; *n* = 5,642 days
**Moderation Variables**^**a,b**^					
**Food Insecurity** [41]	At baseline or follow-up survey	In the last 12 months, did you (or other adults in your household) ever cut the size of your meals or skip meals because there wasn't enough money for food?	Yes/No	A household was categorized as food secure if they had 0 or 1 affirmative responses to these 6 questions and households were classified as food insecure if they answered yes to two or more questions. Almost every month and some months but not every month was coded as an affirmative response to the one frequency question. Often or sometimes true was coded as an affirmative response to the last two questions	26.7% of households were food insecure; *n* = 618 households 14.2% of respondents/ adults cut the size or skipped meals; *n* = 618 households
		How often did this happen?	Almost every month | Some months but not every month | Only 1 or 2 months		69.3% of households that cut the size or skipped meals did so more than only 1 or 2 months; *n* = 88 households
		In the last 12 months, did you ever eat less than you felt you should because there wasn't enough money to buy food?	Yes/No		17.2% of households; *n* = 618 households
		In the last 12 months, were you ever hungry but didn't eat because you couldn't afford enough food?	Yes/No		11.2% of households; *n* = 618
		In the last 12 months, the food that we bought just didn't last, and we didn't have money to get more	Often true | Sometimes true | Never true		29.1% of households report sometimes or often; *n* = 618
		In the last 12 months, we couldn't afford to eat balanced meals	Often true | Sometimes true | Never true		29.3% of households report sometimes or often; *n* = 618
**Household race/ethnicity** [32]	At recruitment, to ensure we had equal representation across race/ethnicity by household type	Please select the race/ethnicity that best characterizes your household (e.g., the foods you cook for your family, the language you speak at home, the holidays you celebrate)	White | Black/African American | Latinx | Hmong | Native American | Somali		28.2% White; 23.5% Black/African-American 13.4% Latinx 15.9% Hmong 16.2% Native American 2.9% Somali
**Race, Parent and child** [42]	At baseline survey	Do you think of yourself/your child as…(you may chose more than one)	White | Black or African American | Hispanic or Latinx | Asian American | Native Hawaiian or other Pacific Islander | American Indian or Native American | Other	Using these two question individuals were categorized as White if race is white only and ethnicity is none; African American if race is Black/African-American only and ethnicity is none; Latinx if race is Hispanic/Latinx only and ethnicity is none; Hmong if race is Asian only and ethnicity is Hmong; Somali/Ethiopian if ethnicity is Somali or Ethiopian; Native American if race is American Indian or Native American only and ethnicity is none; Multi-racial if more than one race was selected or if race selected does not match household race/ethnicity	27.2% children and 27.5% parents were White 17.5% children and 18.6% parents were African American 9.5% children and parents were Latinx 15.0% children and 15.2% parents were Hmong 2.4% children and 2.3% parents were Somali/Ethiopian 8.9% children and 11.7% parents were Native American 19.4% children and 15.2% parents were Multiracial
**Ethnicity, Parent and child** [42]		Is your/your child’s background any of the following?	Hmong | Cambodian | Vietnamese | Laotian | Somali | Ethiopian | Other | None of the above		
**Child sex** [42]	At recruitment	Does your child in the study identify as…	Female | Male		52.4% of children are female

### Sample demographics

The baseline EMA subsample included 631 families who were equally distributed across the six racial/ethnic groups recruited in the study. Primary caregivers, who were mostly mothers from low income households, reported on one study child (mean age at baseline = 6.9; sd = 1.4; see Table 2). Of the 631 families, 618 contributed at least one observation day to this study (i.e., had both a morning observation before noon and an evening meal observation after 4 pm on the same day). The 618 families were observed on 5,642 days.

**Table 2:** Baseline Characteristics of the *Family Matters* EMA Sub-sample (*n* = 618)

Individual	Child	Parent
**Age in years (sd)**	6.9 (1.4)	35.6 (7.1)
**Female (%)**	52.4	91.9
**BMI Percentile (%)**		
5–84	68.6	
85–94	14.1	
95 +	17.3	
**Body Mass Index (kg/m**^**2**^**) (%)**		
< 25		30.4
25–29		28.2
30 +		41.4
**Race/Ethnicity (%)**		
White	27.2	27.5
African American	17.5	18.6
Latinx	9.5	9.5
Hmong	15.0	15.2
Somali/Ethiopian	2.4	2.3
Native American	8.9	11.7
Multiracial	19.4	15.2
**Educational Attainment (%)**		
Some high school		8.7
High school		20.9
Some college		34.0
BA/BS		17.8
Graduate School		18.6
**Household**		
**Number of children (sd)**	3.8 (1.4)	
**Income Level (%)**		
< $20,000	21.8	
$20,000—$34,999	23.1	
$35,000—$49,999	15.7	
$50,000—$99,999	20.9	
$100,000 +	18.4	
**Food Insecure (%)**	26.7	

Unweighted means and percentages

### Measures

All exposure, outcome, and mediation variables used in analyses are presented in Table 1 [32, 34–42] Parent stress, sources of stress, depressed mood and depressive symptoms were ascertained at the signal-contingent survey completed before noon. The food parenting practices, foods served, and child food fussiness measures were obtained at the end-of-day surveys completed after 4 pm. The temporal ordering of the exposure variables several hours before the outcomes serve to avoid reverse causality (e.g., serving fast food for dinner leads to depressed mood).

### Statistical analysis

Inferential statistics were used to examine how morning stress, sources of stress, depressed mood, and depressive symptoms were related to evening food-related parenting practices (pressure-to-eat, restriction, instrumental), food types served at evening dinner (homemade, pre-prepared, and fast food), and child food fussiness. The four predictor variables (stress, sources of stress, depressed mood, and depressive symptoms before noon) were analyzed as continuous random variables. The outcome variables were evaluated using generalized estimating equations with a binomial variance family and logistic link to account for multiple daily observations at two time points, 18-months apart for each family/child. Robust standard errors were used and the covariance structure was set to independent. To adjust for varying numbers of observation days contributed per participant (the average number of days contributed was 9, but ranged from 1 to 16), participant weights were created to give more weight to observations from participants who contributed fewer days. These weights were used in all regression models.

All models were adjusted for household race/ethnicity; whether the observation day occurred on a weekday or weekend, was a part of the baseline or follow-up data collection time point, and whether the day occurred after March 15, 2020 when COVID-19 began to change the daily lives of families in Minnesota; the primary caregiver’s age, sex, weight status, and education; the child’s age, sex, weight status, and multiracial status; and the family’s number of children, income, and food security status at that time point. Because of the large sample, we were able to test for differences in the associations by race/ethnicity, child sex, and food insecurity status with interaction terms. All analyses were performed in Stata 17.0 SE (College Station, TX). We followed the strengthening the reporting of observational studies in epidemiology (STROBE) guidelines for cohort studies in reporting our results (Additional file 1).

## Results

### Descriptive results of parental stress, depressed mood, depressive symptoms, food parenting practices, and child eating behaviors by race/ethnicity, child sex, and food security status

The overall sample morning average stress rating of 1.20 and sources of stress score of 1.07 were higher than the average morning depressed mood rating of 0.74 and the average number of depressive symptoms of 0.35 (see Table 1). All of these morning reports varied significantly by race/ethnicity and food security status, but fewer morning reports varied by child sex (see Table 3, left panel). For example, White parents reported the highest level of stress, African American parents had the highest depressed mood rating and the highest number of depressive symptoms. Hmong parents had the lowest ratings of stress and depressed mood, Somali/Ethiopian parents had the fewest sources of stress and Latinx parents had the lowest depressive symptoms count. Parents of girls rated their stress and depressed mood higher than parents of boys. Finally, parents experiencing food insecurity had higher scores on all four measures compared to food secure parents.

**Table 3:** Descriptive Morning EMA Reports and Evening Meal Behaviors by Race/Ethnicity, Sex, and Food Security Status (*n* = 5,642 person-days)

	**Morning EMA Reports** (range 0–5)				**Evening Meal Behaviors** (yes/no)						
	Stress	Sources of Stress	Depressed Mood	Depressive Sym-ptoms	Pressure-to-eat food parenting	Restrictive food parenting	Instrumental food parenitng	Fast food meal	Pre-prepared meal	Home-made meal	Child food fussiness
	mean (sd)							percent			
**Race/Ethnicity**											
White (ref)	1.46 (1.14)	1.24 (1.08)	0.74 (0.93)	0.21 (0.62)	27.6	3.6	13.4	12.0	10.1	67.8	12.4
African American	**1.18** (1.54)	**0.96** (1.28)	**0.89** (1.37)	**0.58** (1.25)	**21.9**	**7.4**	12.4	**19.3**	**24.1**	**48.4**	**4.5**
Latinx	**0.98** (1.38)	**0.76** (0.96)	**0.61** (1.18)	0.29 (0.90)	**33.8**	**6.8**	15.4	15.4	9.7	70.5	**6.5**
Hmong	**0.79** (1.21)	**0.76** (1.14)	**0.53** (1.05)	**0.33** (1.01)	27.2	**7.4**	12.7	12.7	**7.0**	**73.9**	**8.8**
Somali/ Ethiopian	**1.02** (1.32)	**0.59** (0.85)	**0.54** (1.05)	0.29 (1.09)	22.9	6.8	**6.8**	10.2	**22.8**	**55.9**	**5.9**
Native American	**1.05** (1.23)	1.11 (1.39)	0.79 (1.21)	**0.41** (0.87)	**16.6**	**9.1**	**7.8**	**16.9**	**14.2**	**59.0**	10.0
Multi-racial	**1.34** (1.41)	1.29 (1.37)	**0.85** (1.25)	**0.42** (1.04)	**20.3**	**7.2**	12.3	**17.5**	**17.7**	**57.6**	**8.6**
**Sex**											
Boys (ref)	1.16 (1.27)	1.07 (1.21)	0.66 (1.07)	0.33 (0.93)	26.1	6.6	14.3	13.3	13.2	64.2	9.9
Girls	**1.25** (1.37)	1.06 (1.21)	**0.82** (1.21)	0.37 (0.95)	**23.8**	5.9	**10.9**	**16.5**	14.4	62.0	**8.2**
**Food Security Status**											
Food Secure (ref)	1.13 (1.23)	0.98 (1.08)	0.63 (1.02)	0.24 (0.73)	24.7	5.8	12.2	13.9	11.6	66.5	9.5
Food Insecure	**1.47** (1.57)	**1.37** (1.53)	**1.13** (1.44)	**0.72** (1.40)	25.7	**7.8**	13.8	**18.4**	**21.5**	**51.2**	**7.5**

Unweighted means and percentages. Bolded values are significantly different from reference category at *p* < 0.05

On average, 24.9% of evening meals involved pressure-to-eat food parenting practices while 6.2% of meals involved restrictive food parenting practices and 12.5% of meals involved instrumental food parenting practices (see Table 1). Pressure-to-eat and instrumental practices varied significantly by race/ethnicity and child sex, but the rates did not vary by food security status (see Table 3). Food restriction rates varied by race/ethnicity and food security status, but not by child sex.

For the full sample and all subgroups, more than half of the observed evening meals were completely homemade [43, 44] (see Table 1). On average 63% of meals were homemade, 14% were pre-prepared/store bought (e.g., boxed mac and cheese, frozen pizza), and 15% were fast food (see Table 1). The rate of serving fast food varied significantly by race/ethnicity, child sex, and food security status (see Table 3).

Regarding child eating behaviors, child food fussiness was reported, on average, at 9% of evening meals (see Table 1). White children had the highest rates of food fussiness (see Table 3). Boys were more often reported to be food fussy at a meal and children in food secure families had higher rates of food fussiness.

### Associations between momentary morning reports of parental stress and depressed mood and food parenting practices at the evening meal

Results showed that a one-unit increase in parental stress earlier in the day was associated with 16% greater odds (95% CI, 7% to 25%; *p* < 0.001; Table 4) of parents engaging in pressuring-to-eat food parenting practices and 13% greater odds (95% CI, 0% to 27%; *p* = 0.045) of engaging in food restriction at the evening meal but had no association with instrumental feeding. A one-unit increase in the sources of stress score, which corresponds to a parent endorsing two additional sources of stress that morning, was associated with 25% greater odds (95% CI, 15% to 36%; *p* < 0.001) of engaging in pressuring-to-eat, 22% greater odds (95% CI, 8% to 39%; *p* = 0.002) of food restriction, and 12% greater odds (95% CI, 2% to 23%; *p* = 0.022) of instrumental feeding at the evening meal. A one-unit increase in the depressed mood rating in the morning was associated with 15% greater odds (95% CI, 5% to 26%; *p* = 0.003) of engaging in pressuring-to-eat and 18% greater odds (95% CI, 2% to 36%; *p* = 0.025) of food restriction at dinner the same night. Furthermore, reporting an additional depressive symptom in the morning was associated with 21% greater odds (95% CI, 2% to 42%; *p* = 0.026) of food restriction at the dinner meal.

**Table 4:** Adjusted Associations between Morning Stress, Sources of Stress, Depressed mood, and Depressive Symptoms and Meal Behaviors (*n* = 5642 days on 618 children)

	Morning Predictors (range 0–5):											
	**Stress**			**Sources of Stress**			**Depressed mood**			**Depressive Symptoms**		
Evening Outcomes (yes/no):	OR	95% CI	*p*-value	OR	95% CI	*p*-value	OR	95% CI	*p*-value	OR	95% CI	*p*-value
**Pressure-to-eat food parenting**	1.16	(1.07—1.25)	**< 0.001**	1.25	(1.15—1.36)	**< 0.001**	1.15	(1.05—1.26)	**0.003**	1.01	(0.89—1.14)	0.890
Race/Ethnicity			0.646			0.994			0.229			0.303
Sex			0.905			0.913			0.841			0.584
Food Security			**0.020**			0.235			0.102			0.491
**Restrictive food parenting**	1.13	(1.00—1.27)	**0.045**	1.22	(1.08—1.39)	**0.002**	1.18	(1.02—1.36)	**0.025**	1.21	(1.02—1.42)	**0.026**
Race/Ethnicity			0.256			0.547			0.191			**0.014**
Sex			0.489			0.588			0.357			0.802
Food Security			**0.037**			**0.033**			0.053			0.089
**Instrumental food parenting**	1.09	(1.00—1.18)	0.057	1.12	(1.02—1.23)	**0.022**	1.05	(0.94—1.16)	0.393	1.04	(0.91—1.18)	0.600
Race/Ethnicity			0.608			0.922			0.435			0.941
Sex			**0.034**			0.296			0.223			0.100
Food Security			0.284			0.790			0.167			0.777
**Fast food**	0.94	(0.88—1.01)	0.101	0.93	(0.86—1.01)	0.075	0.92	(0.84—1.00)	**0.040**	1.05	(0.96—1.15)	0.318
Race/Ethnicity			0.165			**0.016**			0.207			0.065
Sex			**0.050**			**0.040**			0.094			**0.007**
Food Security			0.080			0.258			0.202			0.693
**Preprepared meal**	1.08	(0.99—1.17)	0.087	1.00	(0.92—1.09)	0.946	1.08	(0.98—1.19)	0.127	0.91	(0.82—1.01)	0.066
Race/Ethnicity			**0.015**			0.302			0.053			0.367
Sex			**0.016**			**0.030**			0.210			0.891
Food Security			0.058			0.213			0.560			0.488
**Homemade meal**	0.99	(0.93—1.05)	0.713	1.07	(1.00—1.14)	0.053	0.98	(0.91—1.06)	0.674	0.98	(0.90—1.06)	0.593
Race/Ethnicity			0.129			0.116			**0.006**			0.557
Sex			**0.024**			**0.023**			0.316			0.161
Food Security			0.908			0.727			0.571			0.754
**Child Food fussiness**	1.18	(1.08—1.29)	**< 0.001**	1.37	(1.24—1.51)	**< 0.001**	1.12	(1.00—1.25)	**0.049**	1.14	(0.99—1.33)	0.078
Race/Ethnicity			0.299			0.708			0.107			**0.018**
Sex			0.784			0.158			0.617			0.507
Food Security			0.313			0.423			0.368			0.261

Race/Ethnicity, Sex, and Food fussiness refer to the child’s characteristics/behaviors. Adjusted generalized estimated equation (GEE) models with weights include the following covariates: primary caregiver and child age, sex, and weight status; child race/ethnicity; income; food security status, number of children; weekend observation; follow-up observation; observation after start of Covid-19 school shutdowns. Interpretation example: A 1-unit increase in morning stress was associated with 16% greater odds of pressure-to-eat feeding practices (OR: 1.16, 95% CI: 1.07 to 1.25; *p* < 0.001) the same evening at dinner, after controlling for covariates. *OR *Odds ratio, *CI *Confidence interval. The lower three p-values in each panel are from tests of whether interactions between the predictor and child race/ethnicity, child sex, and food security status were significantly different by subgroup

Bolded values are significant at *p* < 0.05

Interaction models indicated that there were significant differences in the associations between morning parental reports of depressive symptoms and evening food restriction by race/ethnicity (see Table 4). We also observed a significant difference in the association between stress and instrumental practices by child sex, and between stress and controlling practices by food security status.

Separate regressions on subgroups indicated that the association between morning stress and food restriction was largely driven by families of White and Latinx children (Table 5). Specifically, a one-unit increase in parental depressive symptoms earlier in the day was associated with 51% greater odds (95% CI, 2% to 122%; *p* = 0.038) of parents of White children, and 98% greater odds (95% CI, 51% to 161%; *p* < 0.001) of parents of Latinx children, engaging in food restriction at the evening meal. Additional analyses shown in Supplemental Table A, which explored sex-specific racial/ethnic differences in associations, revealed that the association between parental depressive symptoms and food restriction among White and Latinx families applied to female children only.

**Table 5:** Adjusted associations for subgroups with significant interactions by race/ethnicity

**Evening Outcome (yes/no)**	**Morning Predictor (range 0–5)**	White	African Am/ Somali/ Ethiopian	Latinx	Hmong	Native American	Multiracial
		OR (95% CI) [*p*-value]					
Restrictive food parenting	Depressive Symptoms	**1.51**	1.25	**1.98**	0.80	0.55	1.24
		**(1.02—2.22)**	(0.94—1.67)	**(1.51—2.61)**	(0.40—1.59)	(0.28—1.05)	(0.99—1.55)
		**[0.038]**	[0.121]	**[< 0.001]**	[0.523]	[0.070]	[0.067]
Fast food	Sources of Stress	0.91	0.92	1.00	1.21	0.82	0.88
		(0.78—1.05)	(0.76—1.10)	(0.71—1.42)	(0.98—1.50)	(0.65—1.03)	(0.78—1.00)
		[0.200]	[0.354]	[0.978]	[0.083]	[0.094]	[0.052]
Preprepared meal	Stress	1.01	**1.17**	0.73	0.99	1.22	0.99
		(0.86—1.17)	**(1.01—1.36)**	(0.52—1.02)	(0.74—1.31)	(0.97—1.54)	(0.86—1.14)
		[0.945]	**[0.038]**	[0.069]	[0.917]	[0.089]	[0.889]
Homemade meal	Depressed Mood	1.02	0.93	1.22	0.99	0.92	1.08
		(0.90—1.15)	(0.78—1.11)	(0.92—1.61)	(0.84—1.17)	(0.71—1.20)	(0.93—1.25)
		[0.756]	[0.432]	[0.172]	[0.920]	[0.541]	[0.319]
Child Food fussiness	Depressive Symptoms	1.29	**1.44**	**2.00**	1.15	1.10	**0.56**
		(0.92—1.81)	**(1.05—1.98)**	**(1.29—3.08)**	(0.90—1.46)	(0.74—1.63)	**(0.35—0.89)**
		[0.135]	**[0.024]**	**[0.002]**	[0.258]	[0.643]	**[0.013]**

Each cell displays results from a separate regression. Adjusted generalized estimated equation (GEE) models with weights include the following covariates: primary caregiver and child age, sex, and weight status; income; food security status, number of children; weekend observation; follow-up observation; observation after start of Covid-19 school shutdowns. Interpretation example: One additional depressive symptom in the morning was associated with 51% higher odds of food restriction among White families (OR: 1.51, 95% CI: 1.02 to 2.22; *p* = 0.038) the same evening at dinner, after controlling for all other covariates in the adjusted models. *OR* Odds ratio, *CI* Confidence interval. Bolded values are significant at *p* < 0.05

The association between parental stress and instrumental feeding occurred among boys, but not girls (Table 6). A one-unit increase in parental stress was associated with 20% greater odds (95% CI, 6% to 36%); *p* = 0.004) of engaging in instrumental feeding if the child was a boy, but no association if the child was a girl.

**Table 6:** Adjusted Associations for Subgroups with Significant Interactions by Child Sex

**Evening Outcome (yes/no)**	**Morning Predictor (range 0–5)**	Boy	Girl
		OR (95% CI) [p-value]	
Instrumental food parenting	Stress	**1.20**	1.00
		**(1.06—1.36)**	(0.90—1.11)
		**[0.004]**	[0.978]
Fast food	Stress	0.99	**0.90**
		(0.89—1.11)	**(0.82—0.98)**
		[0.925]	**[0.021]**
Fast food	Sources of Stress	0.97	**0.89**
		(0.87—1.09)	**(0.81—0.98)**
		[0.623]	**[0.017]**
Fast food	Depressive Symptoms	**1.14**	0.94
		**(1.03—1.27)**	(0.83—1.07)
		**[0.012]**	[0.363]
Preprepared meal	Stress	**1.22**	0.98
		**(1.08—1.37)**	(0.89—1.09)
		**[0.001]**	[0.761]
Preprepared meal	Sources of Stress	1.11	0.92
		(0.99—1.24)	(0.83—1.03)
		[0.086]	[0.141]
Homemade meal	Stress	0.93	1.04
		(0.84—1.01)	(0.97—1.13)
		[0.099]	[0.277]
Homemade meal	Sources of Stress	1.01	**1.12**
		(0.91—1.11)	**(1.03—1.22)**
		[0.920]	**[0.008]**

Each cell displays results from a separate regression. Adjusted generalized estimated equation (GEE) models with weights include the following covariates: primary caregiver and child age and weight status; child race/ethnicity; income; food security status, number of children; weekend observation; follow-up observation; observation after start of Covid-19 school shutdowns. Interpretation example: One additional unit high stress rating in the morning was associated with 20% higher odds of instrumental food parenting practices with boys (OR: 1.20, 95% CI: 1.06 to 1.36; *p* = 0.004) the same evening at dinner, after controlling for all other covariates in the adjusted models. *OR* Odds ratio, *CI* Confidence interval. Bolded values are significant at *p* < 0.05

The associations between morning stress and sources of stress and pressuring-to-eat and food restriction were significant for food insecure families only (see Table 7). For food insecure families, one-unit increase in parental stress was associated with 29% greater odds (95% CI, 14% to 46%; *p* < 0.001) of pressuring-to-eat and 24% greater odds (95% CI, 2% to 52%; *p* = 0.034) of food restriction at the evening meal. Similarly, more sources of stress among food insecure parents was associated with 48% greater odds (95% CI, 20% to 82%; *p* < 0.001) of food restriction at the evening meal.

**Table 7:** Adjusted Associations for Subgroups with Significant Interactions by Food Security Status

**Evening Outcome (yes/no)**	**Morning Predictor (range 0–5)**	Food Secure	Food Insecure
		OR (95% CI) [p-value]	
Pressure-to-eat food parenting	Stress	1.07	**1.29**
		(0.99—1.17)	**(1.14—1.46)**
		[0.089]	**[< 0.001]**
Restrictive food parenting	Stress	0.98	**1.24**
		(0.86—1.12)	**(1.02—1.52)**
		[0.816]	**[0.034]**
Restrictive food parenting	Sources of Stress	1.05	**1.48**
		(0.90—1.21)	**(1.20—1.82)**
		[0.550]	**[< 0.001]**

Each cell displays results from a separate regression. Adjusted generalized estimated equation (GEE) models include the following covariates: primary caregiver and child age, sex, and weight status; child race/ethnicity; income; number of children; weekend observation; follow-up observation; observation after start of Covid-19 school shutdowns. Interpretation example: One additional unit high stress rating in the morning was associated with 29% higher odds of pressuring to eat among food insecure families (OR: 1.29, 95% CI: 1.24 to 1.52; *p* < 0.001) the same evening at dinner, after controlling for all other covariates in the adjusted models. *OR* Odds ratio, *CI* Confidence interval. Bolded values are significant at *p* < 0.05

### Associations between momentary morning reports of parental stress and depressed mood and types of food served at the evening meal

Results showed only one association between morning reports and types of food served at dinner the same day in analyses using the full sample; a higher rating of depressed mood in the morning was associated with 8% lower odds (95% CI, 0.84 to 1.00; *p* = 0.040; Table 4) of having fast food for dinner. However, interaction models indicated that there were numerous significant differences in the associations between morning predictors and the types of foods served in the evening by race/ethnicity and child sex (see Table 4).

Separate regressions by race/ethnicity revealed that for African American and Somali/Ethiopian families (who were combined because of the small number of Somali/Ethiopian observations), a one-unit higher rating of morning stress by parents was associated with 17% greater odds (95% CI, 1% to 36%; *p* = 0.038) of serving a preprepared meal for dinner. While there are significant racial/ethnic differences in the associations between parent’s sources of stress and serving fast food and between parent’s depressed mood and serving a homemade meal, these associations were not significantly different from zero for any racial/ethnic group. Sex-specific racial/ethnic differences in associations, shown in Supplemental Table A, revealed that the association between parent’s sources of stress and serving fast food was significant for female children in African American/Somali/Ethiopian families (OR = 0.74, 95% CI, 0.61 to 0.90; *p* = 0.002) and multiracial families (OR = 0.82, 95% CI, 0.70 to 0.95; *p* = 0.013), and that association between parent’s depressed mood and serving a homemade meal was significant for male children in African American/Somali/Ethiopian families (OR = 0.74, 95% CI, 0.57 to 0.95; *p* = 0.018). In addition, there was a significant association between depressive symptoms and serving fast food to male children in Hmong families (OR = 1.28, 95% CI, 1.02 to 1.61; *p* = 0.030).

Separate regressions by child sex indicated that parents served more fast food and preprepared meals to boys when stressed or depressed, but served less fast food and more homemade meals to girls when stressed (Table 6). Specifically, an additional depressive symptom was associated with 14% greater odds (95% CI, 3% to 27%; *p* = 0.012) of serving fast-food meals if the child was a boy, rather than a girl. A higher stress rating was associated with 22% greater odds (95% CI, 8% to 37%; *p* = 0.001) of serving a preprepared meal if the child was a boy, rather than a girl. A higher stress rating was associated with 10% lower odds (95% CI, 0.82 to 0.98; *p* = 0.021) of serving fast food if the child was a girl, but not if they were a boy. More sources of stress were associated with 11% lower odds (95% CI, 0.81 to 0.98; *p* = 0.017) of serving fast food and 12% greater odds (95% CI, 3% to 22%; *p* = 0.008) of serving a homemade meal if the child was a girl, but if they were a  boy.

### Associations between momentary morning reports of parental stress and depressed mood and child eating behaviors

Results indicated that a one-unit increase in parental stress, sources of stress, and depressed mood earlier in the day was associated with 18% (95% CI, 8% to 29%; *p* < 0.001), 37% (95% CI, 24% to 51%; *p* < 0.001), and 12% (95% CI, 0% to 25%; *p* = 0.049) greater odds of child food fussiness at the evening meal respectively (see Table 4). These three associations did not vary significantly by race/ethnicity, child sex, or food security status. However, the association between parental depressive symptoms and child food fussiness varied significantly by race/ethnicity. An additional depressive symptom in the morning was associated with 44% greater odds (95% CI, 5% to 98%; *p* = 0.024) of food fussiness in African American/Somali/Ethiopian families, 100% greater odds (95% CI, 29% to 208%; *p* = 0.002) of food fussiness in Latinx families, and 44% lower odds (95% CI, 0.35 to 0.89; *p* = 0.013) of food fussiness among multiracial families. Sex-specific racial/ethnic differences in associations, shown in Supplemental Table A, revealed that these associations were among female children in these three racial/ethnic group, but not male children.

## Discussion

Our findings support and extend the limited existing research in the field regarding the relationships between stress, depressed mood/symptoms, food parenting, and child eating behaviors. First, our results support prior study findings by showing that parental experiences of stress and depressed mood earlier in the day were associated with increased risk for pressure-to-eat food parenting [19] and that households experiencing food insecurity were more likely to engage in controlling food parenting practices the same day at the evening meal [45] Controlling food parenting practices are known to be associated with increased risk for overweight and obesity in children, which are important predictors of future CVD [46–49] and thus would be important to mitigate.

Second, our findings extend prior research by showing significant associations between parental experiences of stress, sources of stress, depressed mood, and depressive symptoms with increased risk of restrictive food parenting practices the same day at the evening meal [19] In addition, our results showed significant associations between parental experiences of stress, sources of stress, and depressed mood, and increased risk for child food fussiness the same day at the evening meal. Both restrictive food parenting and child food fussiness are important to reduce, given they have both been shown in prior research to be associated with increased risk for overweight and obesity in children [50, 51].

Our findings also extend prior research by showing associations between parental experiences of stress, sources of stress, depressed mood, and depressive symptoms earlier in the day with food parenting practices and child eating behaviors the same day at the evening meal were dependent on race/ethnicity and child sex. Morning stress and depressed mood and food parenting practices appear to be most strongly associated for girls. Specifically, parental experiences of depressive symptoms in the morning and associations with restrictive food parenting at the evening meal were strongest for parents of White and Latinx girls. Parental experiences of depressive symptoms in the morning and child food fussiness at the evening meal were strongly positively associated for parents of African American/Somali/Ethiopian and Latinx girls, and strongly negatively associated for parents of Multiracial girls. In contrast, the associations between parental stress and depressed mood and the types of foods served were significant for both boys and girls, but in opposite directions. Parental reports of more stress and depressive symptoms were associated with a higher likelihood of serving fast food and preprepared meals if the child was a boy, while more stress and sources of stress were associated with a lower likelihood of serving fast food and a higher likelihood of serving homemade meals if the child was a girl. These sex-specific results provide important information for health care clincians and researchers with regard to key messages to share with parents dependent on their child’s sex. In addition, sex-specific results merit future qualitative research in order to better understand potential causal mechanisms of these findings.

Study results have several implications for health care clincians, researchers, and interventionist in the US and internationally. For health care settings, clinicians may want to consider screening parents for stress and depressive symptoms, or continue to if they already are, during well-child visits to provide potential resources and anticipatory guidance to parents about the increased risk of engaging in controlling food parenting practices in the face of high psychological distress. It may also be helpful for providers to offer resources to parents regarding stress reduction (e.g., apps, referrals to mental health providers, online resources) and educate parents during well-child visits regarding the momentary influences that stress and depressed mood can have on food parenting practices and child eating behaviors. Many parents may be unaware that their stress levels or depressed mood could influence their food parenting practices or their child’s eating behaviors. It is important for providers to use respectful, supportive, and culturally appropriate language when delivering these messages to parents, while keeping in mind the larger structural and socioeconomic barriers that might be driving parents’ stress levels and consequent parenting practices and child behaviors.

For researchers, it may be important to consider developing interventions using momentary intervention techniques such as ecological momentary intervention (EMI) to intervene in real-time with parents to help them engage in healthful food parenting practices when experiencing high psychological distress. Furthermore, intervention researchers may want to consider tailoring interventions with specific populations, such as Multiracial households, Latinx households, and households with sons to address stress and depressed mood in order to increase healthful food parenting practices. 

There were both strengths and limitations of the current study. Strengths of the current study include the use of EMA to measure behaviors within and across days over a seven-day period and across two-time points, 18 months apart. Additionally, EMA was used to measure both exposure and outcome variables across time and context. Furthermore, the sample included racially/ethnically diverse households. There were also limitations of the current study, including: (a) use of some survey items that have not been validated for EMA and that are individual items rather than scales; (b) the population was drawn from one geographic location; thus, generalizing findings to other populations should be done cautiously; (c) this study only examined the relationships between parental stress/depressed mood and food parenting practices and child eating behaviors, it is also possible that child eating behaviors may lead to parental stress or depressed mood. It would be important to examine this bidirectional relationship in future research.

## Conclusion

Study results indicated that high parental stress, sources of stress, depressive symptoms, and depressed mood earlier in the day predicted pressure-to-eat and restrictive food parenting practices, and food fussiness in children. Some of the associations varied by race/ethnicity, child sex, and food security status. Recommendations for future research include developing interventions using EMI to target momentary factors such as stress and depressive symptoms to promote healthful food-related parenting practices. Health care clinicians may also want to use study findings to guide their anticipatory guidance with parents during well-child visits regarding the influence that stress and depressed mood can have on everyday food parenting practices.

## Supplementary Information

**Additional file 1.** STROBE Statement—Checklist of items that should be included in reports of cohort studies.

**Additional file 2:** **Supplemental Table A.** Adjusted Associations for Subgroups with Significant Interactions by Sex & Race/Ethnicity.

## Data Availability

Data used in the current study are available and may be obtained from the corresponding author upon reasonable request.

## Abbreviations

EMA: Ecological momentary assessment
EMI: Ecological momentary intervention
