# Supplementary material for: Real-time predictors of food parenting practices and child eating behaviors in racially/ethnically diverse families
Source: Int J Behav Nutr Phys Act. 2023 Jul 11;20:86. doi: 10.1186/s12966-023-01476-4 (PMC10337081; doi:10.1186/s12966-023-01476-4)
Supplement: Supplementary file 2 [file 12966_2023_1476_MOESM2_ESM.docx]

Supplemental Table A:  Adjusted Associations for Subgroups with Significant Interactions by Sex & Race/Ethnicity 

| Evening Outcome | Restrictive food parenting | Fast Food | Fast Food | Homemade meal | Food Fussiness |
| --- | --- | --- | --- | --- | --- |
| Morning Predictor | Depressive Symptoms | Sources of Stress | Depressive Symptoms | Depressed Mood | Depressive Symptoms |
|  | OR (95% CI) [p-value] |
| White Male | 0.78 | 0.85 | 1.11 | 1.14 | 1.48 |
| (0.27 - 2.28) | (0.70 - 1.03) | (0.76 - 1.63) | (0.92 - 1.40) | (0.96 - 2.28) |
| [0.654] | [0.099] | [0.581] | [0.233] | [0.076] |
| White Female | 2.06 | 0.95 | 1.16 | 1.00 | 1.61 |
| (1.33 - 3.19) | (0.75 - 1.20) | (0.87 - 1.55) | (0.85 - 1.18) | (0.99 - 2.61) |
| [0.001] | [0.662] | [0.326] | [0.999] | [0.057] |
| Af Am/ Somali/ Ethiopian Male | 1.22 | 1.08 | 1.09 | 0.74 | 1.21 |
| (0.76 - 1.96) | (0.85 - 1.37) | (0.86 - 1.39) | (0.57 - 0.95) | (0.87 - 1.68) |
| [0.415] | [0.548] | [0.462] | [0.018] | [0.258] |
| Af Am/ Somali/ Ethiopian Female | 1.33 | 0.74 | 1.01 | 1.20 | 1.78 |
| (0.82 - 2.15) | (0.61 - 0.90) | (0.80 - 1.28) | (0.98 - 1.47) | (1.05 - 3.00) |
| [0.241] | [0.002] | [0.914] | [0.074] | [0.031] |
| Latinx Male | 1.54 | 0.83 | 1.26 | 1.12 | 1.34 |
| (0.74 - 3.24) | (0.37 - 1.88) | (0.87 - 1.82) | (0.80 - 1.57) | (0.68 - 2.62) |
| [0.251] | [0.663] | [0.228] | [0.513] | [0.401] |
| Latinx Female | 2.17 | 1.03 | 1.00 | 1.16 | 19.02 |
| (1.18 - 3.99) | (0.74 - 1.45) | (0.60 - 1.66) | (0.85 - 1.57) | (4.10 - 88.30) |
| [0.012] | [0.843] | [0.999] | [0.351] | [<0.001] |
| Hmong Male | 0.68 | 1.12 | 1.28 | 1.16 | 1.21 |
| (0.46 - 1.02) | (0.82 - 1.54) | (1.02 - 1.61) | (0.92 - 1.47) | (0.91 - 1.60) |
| [0.061] | [0.477] | [0.030] | [0.220] | [0.190] |
| Hmong Female |  | 1.25 | 1.10 | 1.00 | 1.12 |
|  | (0.93 - 1.66) | (0.89 - 1.36) | (0.80 - 1.24) | (0.59 - 2.15) |
|  | [0.135] | [0.387] | [0.995] | [0.726] |
| Native American Male |  | 0.67 | 1.53 | 1.17 | 2.00 |
|  | (0.40 - 1.14) | (0.73 - 3.19) | (0.70 - 1.96) | (0.27 - 14.77) |
|  | [0.143] | [0.256] | [0.551] | [0.497] |
| Native American Female | 0.71 | 0.85 | 0.95 | 0.81 | 1.12 |
| (0.34 - 1.47) | (0.64 - 1.15) | (0.66 - 1.36) | (0.56 - 1.18) | (0.79 - 1.59) |
| [0.355] | [0.295] | [0.778] | [0.271] | [0.516] |
| Multiracial Male | 1.40 | 1.00 | 1.11 | 1.19 | 0.63 |
| (0.90 - 2.18) | (0.82 - 1.23) | (0.94 - 1.32) | (0.96 - 1.47) | (0.35 - 1.16) |
| [0.139] | [0.973] | [0.203] | [0.107] | [0.137] |
| Multiracial Female | 1.22 | 0.82 | 0.82 | 1.07 | 0.48 |
| (0.86 - 1.72) | (0.70 - 0.96) | (0.63 - 1.07) | (0.89 - 1.28) | (0.26 - 0.88) |
| [0.269] | [0.013] | [0.139] | [0.484] | [0.018] |

Adjusted generalized estimated equation (GEE) models with weights include the following covariates: primary caregiver and child age and weight status; income; food security status, number of children; weekend observation; follow-up observation; observation after start of Covid-19 school shutdowns. Interpretation example:  An additional depressive symptom in the morning was associated with 106% greater odds of food restriction of girls in White families (OR: 2.06, 95% CI: 1.33 to 3.19; p=0.001) the same evening at dinner, after controlling for covariates. OR=odds ratio; CI=confidence interval. Blank cells indicate models that would not converge due to lack of variation in the small sub-sample.

Bolded values are significant at p <0.05.
